# Trematodes in snail intermediate hosts of a subarctic lake: updated perspectives on molecular diversity

**DOI:** 10.1016/j.ijppaw.2026.101236

**Published:** 2026-05-14

**Authors:** Michal Benovics, Camila Pantoja, Petra Kundid, Christian Selbach, Miroslava Soldánová

**Affiliations:** a Department of Zoology, Faculty of Natural Sciences, Comenius University in Bratislava, Ilkovičova 6, Bratislava, 842 15, Slovakia; b Unit for Environmental Sciences and Management, North-West University, Potchefstroom, 2520, South Africa; c Institute of Parasitology, Biology Centre of the Czech Academy of Sciences, České Budějovice, Czech Republic; d Department of Arctic and Marine Biology, Faculty of Biosciences, Fisheries and Economics, UiT the Arctic University of Norway, Tromsø, Norway

**Keywords:** Aquatic parasites, Molluscs, Intermediate hosts, Community turnover, Phylogeny, Lake Takvatn

## Abstract

Subarctic freshwater ecosystems have traditionally been considered species-poor in parasites, but recent molecular studies increasingly challenge this view. We present an updated molecular assessment of trematode diversity in molluscan intermediate hosts from Lake Takvatn, northern Norway, more than a decade after the first comprehensive survey (2012–2013) of this system. A total of 2496 molluscs representing five species were examined in 2024. Molecular analyses of intramolluscan stages revealed 24 trematode species and species-level lineages belonging to seven families. Trematode prevalence in first intermediate hosts reached 12.3% and was higher in *Ampullaceana balthica* (18.1%) than in *Gyraulus acronicus* (2.3%), while only a few infections were detected in other molluscan hosts (ten in sphaeriids and one in *Valvata* sp.). Molluscs also frequently served as second intermediate hosts, particularly for *Cotylurus cornutus* and *Echinoparyphium recurvatum*. Despite comparable overall species richness between surveys (both studies reporting 24 taxa), community composition showed apparent turnover, with 15 taxa persisting across surveys, while nine previously recorded taxa were not detected and nine newly recorded lineages were identified, including several potentially new to science. The consistent representation of trematode families between the two surveys indicates long-term structural persistence despite species-level turnover. *Ampullaceana balthica* remained the main host for trematode transmission, hosting 71% of detected taxa, whereas *G. acronicus*, previously considered of minor importance, emerged as a significant first and second intermediate host for multiple species/lineages. Our results demonstrate that subarctic lakes can sustain relatively high trematode species richness despite low host diversity, and that trematode assemblages may exhibit temporal changes in composition rather than directional loss of diversity. Host availability, particularly of definitive bird hosts, appears to be a key factor maintaining trematode transmission across high-latitude freshwater ecosystems. These findings highlight the value of snapshot resampling approaches for assessing temporal dynamics of parasite communities in natural systems.

## Highlights

• Molecular data revealed 24 trematode taxa parasitising molluscs in a subarctic lake.
• Decadal comparison shows trematode turnover but stable family structure.
• *Ampullaceana balthica* hosted most trematode taxa.
• *Gyraulus acronicus* hosts multiple previously undetected lineages.
• Low-prevalence trematode taxa detected using integrative approach.

## Graphical abstract

**Figure:**
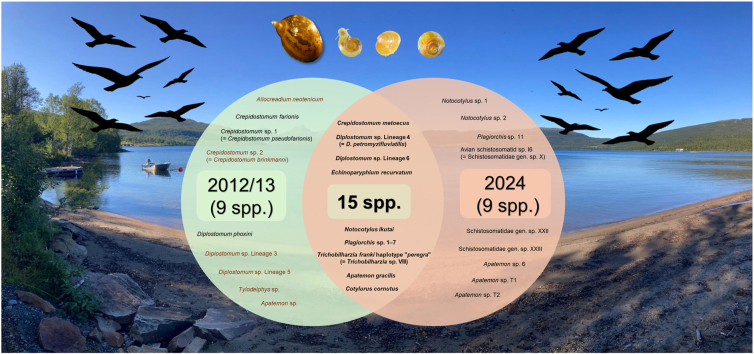

## 1 Introduction

Digenean trematodes are among the most diverse groups of metazoan parasites and represent a major component of global parasite diversity in aquatic environments (Cribb et al., 2001; Pérez-Ponce de León, 2001; Esch et al., 2002; Littlewood et al., 2015). Beyond their taxonomic richness, trematodes play key roles in host population dynamics and food-web structure, influencing ecological interactions, energy flow and biomass distribution (Kuris et al., 2008; Lafferty et al., 2008; Preston et al., 2013; Frainer et al., 2018; Nadler et al., 2023). This ecological importance largely stems from their complex life cycles, which link multiple hosts across trophic levels and ecosystem boundaries.

Molluscs (predominantly gastropods) are central to these life cycles as obligatory first intermediate hosts, followed by transmission to invertebrate and vertebrate second intermediate and definitive hosts (Esch et al., 2002; Galaktionov and Dobrovolskij, 2003). Within snails, trematodes reproduce asexually (via sporocysts and/or rediae) and continuously generate large numbers of free-living infective stages, the cercariae, which in many species actively seek subsequent hosts. This makes snail communities key reservoirs of trematode diversity (Sures et al., 2025) and valuable indicators of transmission pathways and ecosystem-level biodiversity (Hechinger and Lafferty, 2005; Hechinger et al., 2007). Snails can also serve as second intermediate hosts for some trematodes, harbouring metacercariae that await trophic transmission, further linking benthic habitats and higher trophic levels. Thus, trematode life cycles connect host availability with broader community interactions and aquatic food-web functioning (Poulin and Morand, 2004; Marcogliese, 2005; Lafferty et al., 2008). This underscores the importance of understanding complete life cycles when interpreting patterns of parasite diversity and transmission (Blasco-Costa and Poulin, 2017).

Understanding these ecological links depends on reliable species-level identification. However, accurate assessment of trematode diversity has long been hindered by interspecific morphological similarity and the presence of cryptic taxa. These limitations are particularly pronounced in larval stages, especially cercariae, where morphology alone often lacks sufficient resolution for reliable species-level identification, despite the presence of diagnostic features for major trematode groups (Hechinger, 2023; Cribb et al., 2025). Molecular approaches have therefore become essential, revealing numerous previously unrecognised lineages in snails (e.g., Georgieva et al., 2013; Kundid et al., 2024) and unexpectedly high trematode richness across aquatic ecosystems, including high-latitude freshwater habitats (e.g., Soldánová et al., 2017, Gordy and Hanington, 2019; Kudlai et al., 2021; Pantoja et al., 2021; Faltýnková et al., 2025 and references therein).

Subarctic freshwater ecosystems provide a distinctive context for studying trematode diversity. Characterised by strong seasonality, long winters, short productive summers and relatively simple host communities (Selbach and Paterson, 2025), these systems were long assumed to support species-poor parasite faunas (Galaktionov, 1996; Dobson et al., 2015; Hoberg et al., 2013; Kutz et al., 2009). However, recent molecular studies demonstrate that subarctic freshwater habitats can sustain diverse trematode assemblages shaped by the dispersal of migratory (mainly bird) hosts and the availability of suitable intermediate hosts (Soldánová et al., 2017; Gordy and Hanington, 2019; Shaw et al., 2020; Faltýnková et al., 2025 and references therein). In such host-limited systems, trematodes may therefore constitute a disproportionately important component of biodiversity and act as sensitive indicators of ecological and environmental change across northern landscapes (reviewed in Faltýnková et al., 2025; Louvard et al., 2025).

Lake Takvatn in northern Norway (69°07′N, 19°05′E; 214 m a.s.l.; surface area 14.2 km^2^), the focus of this study, is an oligotrophic lake and one of the best-studied freshwater ecosystems in the subarctic, serving as a long-term model system for ecological and parasitological research (Amundsen et al., 2009, 2019). The only community-level molecular survey of trematodes in this lake revealed exceptionally high diversity, with 24 species and genetic lineages detected across various aquatic hosts (Soldánová et al., 2017). Most of this diversity was associated with molluscs, particularly *Ampullaceana balthica* (formerly *Radix balthica*; Lymnaeidae), identified as a key first intermediate host compatible with 19 trematode taxa. In contrast, infections in the second most abundant snail, *Gyraulus acronicus* (Planorbidae) were rare and recorded only as metacercariae, leaving its role as a first intermediate host uncertain, as no intramolluscan stages were previously detected in this species (Soldánová et al., 2017), despite its known potential to host trematodes. Subsequent molecular research in Takvatn has focused on avian schistosomes, confirming one species, *Trichobilharzia* sp. “peregra” (Soldánová et al., 2022), and identifying three additional schistosome lineages (i.e., a genetically divergent monophyletic sequence clusters non-congeneric with *Trichobilharzia*), two of which are novel records, including the first detection of two lineages in *G*. *acronicus* from a subarctic ecosystem (Kibet et al., in press). Together, these studies indicate that at least 27 trematode taxa infect molluscs in Takvatn and provide a molecular reference framework for future comparisons of trematode diversity at high latitudes. Further studies in Takvatn have shown that trematodes contribute substantially to parasite diversity and strongly influence food-web structure and energy pathways in the lake ecosystem (Born-Torrijos et al., 2020, 2021; Shaw et al., 2020; Moore et al., 2024), supporting broader evidence that parasites are integral components of aquatic ecosystems (Kuris et al., 2008; Lafferty et al., 2008).

Despite increasing research efforts, trematode diversity in snails and its temporal persistence remain poorly understood, as species inventories are still incomplete in many geographic regions, including northern ecosystems (reviewed in Faltýnková et al., 2025), and often overlook molluscan hosts despite their central role in trematode life cycles (Esch et al., 2002). Subarctic ecosystems also experience pronounced environmental variability and ongoing climate change (Kutz et al., 2009; Hoberg et al., 2012; Galaktionov, 2017; Amundsen et al., 2019; Morison et al., 2023), including shifts in ice cover and temperature regimes. These changes can alter host availability and the seasonal transmission window, thus influencing parasite assemblages and transmission dynamics (Marcogliese, 2005; Galaktionov, 2016; Galaktionov et al., 2019; Selbach et al., 2024). Because long-term assessments remain scarce and often focus on changes in parasite infection levels rather than changes in diversity (Hammoud et al., 2026), snapshot resampling of trematode diversity after extended time gaps therefore offers a valuable opportunity to evaluate the stability and temporal dynamics of parasite assemblages in subarctic ecosystems, especially where molecular baseline data already exist.

In this study, we present an updated assessment of trematode molecular diversity in Lake Takvatn, Norway, focusing on infections in freshwater molluscan intermediate hosts. We examined several gastropod species, with emphasis on *Ampullaceana balthica* (Lymnaeidae) and *Gyraulus acronicus* (Planorbidae), the two common and ecologically important snail species in the lake. Our research is based on material collected in July, August and October 2024, more than a decade after the initial molecular survey by Soldánová et al. (2017). In particular, *A. balthica* is widely recognised as an important first intermediate host of diverse trematodes across Europe (Duan et al., 2021; Schwelm et al., 2021), including high-latitude regions (Faltýnková et al., 2025), whereas the trematode fauna of *G. acronicus* remains poorly explored (Faltýnková et al., 2016). Using molecular identification of intramolluscan stages (sporocysts, rediae and cercariae) and, where present, metacercariae indicating the role of molluscs as second intermediate hosts, we aimed to (i) reassess trematode species richness after more than ten years, (ii) evaluate the persistence and turnover of trematode communities, and (iii) further elucidate the ecological role of the two snail species as key transmission hubs in a subarctic lake ecosystem.

## 2 Materials and methods

### 2.1 Sampling and material processing

The molluscan community of Lake Takvatn comprises five species: three gastropods, *Ampullaceana balthica* (Lymnaeidae), *Gyraulus acronicus* (Planorbiidae) and *Valvata* sp. (Valvatidae), and two small clams, *Pisidium casertanum* and *Sphaerium* sp. (Bivalvia, Sphaeriidae) (Soldánová et al., 2017; present study). The main sampling focus was on the snails *A. balthica* and *G. acronicus*, which were the most frequently encountered and most readily sampled molluscan hosts in the littoral zone, while less abundant or less accessible taxa such as *Valvata* sp. and sphaeriid clams were collected opportunistically when encountered. Sampling effort was therefore not equal across all host species, but reflected their relative availability and detectability in the field. This sampling strategy reflects the structure of the littoral molluscan community and their known importance as hosts in this system (see Soldánová et al., 2017). Host identity followed previous molecular confirmation for *A. balthica* (syn. *Radix balthica*) and sphaeriid bivalves (Soldánová et al., 2017) and for *G. acronicus* (Kibet et al., in press). The identity of *Valvata* specimens, previously identified only morphologically as *Valvata piscinalis* (Soldánová et al., 2017), was verified molecularly in this study using tissue samples preserved in 96% molecular-grade ethanol.

Sampling was conducted in July, August and October 2024 at three littoral sites of the lake (69°05′12.2″N, 19°08′17.6″E; 69°05′40.7″N, 19°06′50.4″E and 69°07′02.9″N, 19°05′03.9″E), the latter two supporting high densities of *A*. *balthica* and *G*. *acronicus*. Snails were collected by hand from stones in shallow water (≤1 m depth) and by dragging a sieve sampler (1 mm mesh size) along the lake bottom from a boat at depths of 2–10 m in the littoral zone. Macrophyte material retained in the sieve was subsequently sorted to recover associated molluscs, including *Valvata* sp. and sphaeriid bivalves. This sampling approach is consistent with the methodology applied in the previous survey of the lake (Soldánová et al., 2017), allowing direct comparison between datasets.

Molluscs were placed individually in beakers with filtered lake water and exposed to light for 48 h at laboratory temperature (approximately 20 °C) to stimulate cercarial emergence. Cercariae were then examined alive and preliminarily identified based on morphological characteristics using standard keys (Faltýnková et al., 2007, 2008). After the exposure period, all molluscs were dissected under a Zeiss Stemi DV4 stereomicroscope (Carl Zeiss Microlmaging GmbH, Göttingen, Germany) to detect sporocysts or rediae (prepatent infections) and metacercariae. Cercariae were photodocumented using a light microscope equipped with a Bresser MicroCam II digital camera (Bresser GmbH, Rhede, Germany).

To further evaluate sampling completeness and account for differences in sampling effort among host species, rarefaction analyses were performed in RStudio 2023.12.1 (Posit Team, 2024; R Core Team, 2024) using the package *iNEXT* (Hsieh et al., 2025), based on incidence (presence-absence) data, with individual snails treated as sampling units. Separate incidence matrices were constructed for each snail host, excluding taxa absent from all samples within a host. Sampling completeness was quantified as sample coverage, and rarefaction and extrapolation curves were used to assess the relationship between sampling effort and species diversity. Ninety-five percent confidence intervals were estimated by bootstrap resampling. Visualisation was performed using the package *ggplot2* (Wickham, 2016).

Both the number of infected host individuals and the total number of infection records were documented, with the latter including double infections within a single host and therefore exceeding the number of infected hosts. Prevalence was calculated at the trematode family level as the proportion of infected host individuals relative to the total number of examined individuals, following Bush et al. (1997). Because not all infections could be reliably identified to the species level, family-level assignments were used to retain all available data and allow consistent comparison across samples. Metacercariae of *Plagiorchis* spp. were occasionally observed in *A*. *balthica* snails simultaneously harbouring sporocysts and cercariae of the same taxon, typically in hosts in poor condition. Because *Plagiorchis* metacercariae were never found in snails lacking intramolluscan stages (sporocysts or rediae), these cases were interpreted as in-host encystment rather than independent infections in second intermediate hosts and were therefore excluded from prevalence estimates. A similar phenomenon was already noted in Soldánová et al. (2017), resulting in some *Plagiorchis* isolates being reported as metacercariae.

All detected infections were preserved in 96% molecular-grade ethanol for DNA analysis. However, not all infections were analysed molecularly, either due to amplification failure or by design when previous studies demonstrated conspecificity. For example, only two isolates of *Trichobilharzia* sp. VIII were sequenced out of 28 infection records and are included in Kibet et al. (in press). This genetic lineage has consistently corresponded to the same species in previous molecular studies from Takvatn, where it was reported as *Trichobilharzia franki* haplotype “peregra” or *Trichobilharzia* sp. “peregra” (Soldánová et al., 2017, 2022). Nevertheless, preliminary morphological identification allowed reliable assignment of infections to the genus level, enabling calculation of family-level prevalence for most taxa. Prepatent infections represented by sporocysts with only germ cells or immature cercariae could not be successfully identified molecularly (Supplementary Table S1) and were therefore excluded from taxon-level prevalence estimates. Only taxa identified to species or putative species-level lineage (as established in the previous publications investigating respective taxa) were included in richness comparisons, and unresolved infections were not treated as separate taxa to ensure consistency and avoid overestimation of species richness. Molecular vouchers were deposited at the Laboratory of Helminthology, Biology Centre of the Czech Academy of Sciences, České Budějovice, Czech Republic.

### 2.2 Genomic DNA extraction, amplification and sequencing

Prior to DNA extraction, pooled cercariae, rediae, sporocysts, or metacercariae stored in 96% molecular-grade ethanol were dried using a vacuum centrifuge. Genomic DNA was then extracted using the DNeasy Blood & Tissue Kit (Qiagen, Hilden, Germany) following the manufacturer's protocol. After DNA extraction, polymerase chain reactions (PCR) were carried out in a total volume of 20 μl containing 10 μl of FIREPol Master Mix Ready to Load (Solis BioDyne OÜ, Tartu, Estonia), 0.5 μM of each primer, 3 μl of DNA template, and nuclease-free water. The selected regions for amplification were selected to be able to compare the newly obtained sequence data to sequences from previous survey (Soldánová et al., 2017), and the respective primers used for each expected trematode genus are presented in the Supplementary Table S2. The species identity of selected *Valvata* (Gastropoda, Valvatidae) specimens was confirmed using primers and the amplification protocol described in Folmer et al. (1994). PCR products were detected by electrophoresis in 1.5% agarose gels stained with GoodView (SBS Genetech, Beijing, China). Amplified products were purified using EPPiC Fast (A&A Biotechnology, Gdansk, Poland), following the manufacturer's protocol. For bidirectional sequencing, services of Macrogen Europe were employed (Amsterdam, Netherlands).

### 2.3 Sequence dataset assembly and phylogenetic analyses

The newly obtained sequences were used to assess the species identity of collected larval stages of trematodes. To determine the phylogenetic position of the collected specimens, additional orthologous sequences from congeners or phylogenetically close species were obtained from GenBank (the list of retrieved sequences is in Supplementary Table S3). The sequences were selected based on their overlapping length and sufficient associated metadata supporting the origin of the sequence specimens.

A total of six sequence datasets were assembled with aim to include the longest available unique congeneric sequences per species/putative species-level genetic lineage as determined in previous studies (i.e., Soldánová et al., 2017; Lebedeva et al., 2022; Izrailskaia et al., 2024; Stanevičiūtė et al., 2025): (i) a dataset for assessing the phylogenetic position and species designation for *Apatemon* species, consisting of partial COI sequences (including *Australapatemon* sp. ortholog as the outgroup for rooting phylogenetic trees); (ii) a dataset encompassing *Cotylurus* species build of partial COI sequences (including *Apharyngostrigea pipientis* ortholog as the outgroup); (iii) a dataset encompassing *Diplostomum* species build of partial COI sequences (including *Tylodelphys aztecae* ortholog as the outgroup); (iv) a dataset including *Echinoparyphium* ortholog ND1 sequences (including *Echinostoma revolutum* ortholog as the outgroup); (v) a dataset encompassing Notocotylidae species COI sequences (including *Hippocrepis hippocrepis* ortholog as the outgroup); and (vi) a dataset including sequences of non-orthologous COI regions of various *Plagiorchis* species (including *Orientocreadium elegans* ortholog as the outgroup). *Plagiorchis* sequences from the previous study of Soldánová et al. (2017) were omitted from the dataset due to their insufficient length, however conspecific sequences available in GenBank were used. The ortholog sequences in each dataset were aligned using the fast Fourier transform algorithm implemented in MAFFT (Katoh, 2002), using the G-INS-i refinement method. Each alignment was treated as codon partitioned, and for subsequent phylogenetic analyses, a GTR model was selected independently for each position within the codon, including both a gamma distribution and the proportion of invariable sites. Phylogenetic trees were constructed using Bayesian inference (BI) and Maximum likelihood (ML) approaches in MrBayes 3.2 (Ronquist et al., 2012) and RAxML 8.1.12 (Stamatakis, 2006; Stamatakis, 2014), respectively. BI analysis used the Metropolis-coupled Markov chain Monte Carlo algorithm with two parallel runs of one cold and three hot chains, and was run for 10^6^ generations (except for *Plagiorchis* and *Diplostomum* datasets, which were run for 2 × 10^6^ and 5 × 10^6^ generations, respectively), sampling trees every 100 generations. The initial 30% of all saved trees were discarded as “burn-in” after checking that the standard deviation split frequency fell below 0.01. The convergence of the runs and the parameters of individual runs were checked using Tracer v. 1.7.1 (Rambaut et al., 2018). Posterior probabilities for each tree node were calculated as the frequency of samples recovering a given clade. Clade bootstrap support for ML trees was assessed by simulating 10^3^ pseudoreplicates.

## 3 Results

A total of 2496 molluscs of five species were examined during three sampling occasions in 2024. Of these, 307 individuals were infected as first intermediate hosts (overall prevalence 12.3%; Table 1). However, a total of 319 infection records (12.8%) were detected, exceeding the number of infected hosts due to double infections recorded exclusively in *A*. *balthica* (n = 12; 0.8% of examined individuals), most commonly involving *Plagiorchis* spp. in combination with other trematode taxa (n = 10). According to the sampling design, which followed the approach of Soldánová et al. (2017), and consistent with previous data from Lake Takvatn, the molluscan fauna was numerically dominated by the two most frequently encountered snail species, *A. balthica* (n = 1589) and *G*. *acronicus* (n = 701) (Table 1). Trematode prevalence was higher in *A. balthica* (18.1%) than in *G. acronicus* (2.3%) (Table 1), while infection levels in *Valvata* sp. and sphaeriid clams remained low (Table 1), as reflected in opportunistic sampling. The contrast in parasite species diversity between the two key snail species is therefore well supported by large sample sizes. Most infection records were successfully identified molecularly (235 of 319; 74%; Table 1), and majority of them corresponded to patent infections with cercarial emergence (Supplementary Table S1). Of the unidentified infections, most originated from *A. balthica* (72 records), including 18 infections represented by prepatent sporocyst infections (1.1%) (Supplementary Table S1). In addition, five infections from *G. acronicus* and one each from *Valvata* sp. and sphaeriid clams could not be assigned molecularly (Table 1).

**Table 1:** Trematode infections and prevalence (%) in molluscan first (sporocysts, rediae and cercariae) and second (metacercariae) intermediate hosts in Lake Takvatn in 2024.

Host family/species	First intermediate host				Second intermediate host	
	Examined	Infected (%)	Infection records^a^ (%)	Molecularly identified	Infected (%)	Molecularly identified
**Gastropoda**						
**Lymnaeidae**						
*Ampullaceana balthica*	1589	287 (18.1)	299 (18.8)	224	181 (11.4)	8
**Planorbidae**						
*Gyraulus acronicus*	701	16 (2.3)	16 (2.3)	9	73 (10.4)	2
**Valvatidae**						
*Valvata* sp.	86	1 (1.2)	1 (1.2)	0	0	0
**Bivalvia**						
**Sphaeriidae**						
*Pisidium casertanum/Sphaerium* sp.	120	3 (2.5)	3 (2.5)	2	7 (5.8)	1
**TOTAL**	2496	307 (12.3)	319 (12.8)	235	261 (10.5)	11

a Total number of detected infections, including double infections within a single host individual.

To assess sampling completeness across host species, sample coverage and rarefaction analyses were conducted (Supplementary Fig. S1). *Ampullaceana balthica* showed high sample coverage (98.6%), indicating that most trematode diversity was captured, whereas *G*. *acronicus* exhibited lower coverage (68.8%) and a high proportion of singleton species, suggesting incomplete sampling. This pattern was consistent with rarefaction analyses: curves for *A. balthica* approached a plateau, while those for *G. acronicus* continued to rise with increased sampling effort, indicating that additional species are likely to be detected with further sampling (Supplementary Fig. S1). Estimates of sampling completeness for *P*. *casertanum*/*Sphaerium* sp*.* and *Valvata* sp. were not reliable due to the extremely low number of detected infections (Supplementary Fig. S1, Table 1), limiting the applicability of coverage-based estimators.

Molluscs also served as second intermediate hosts, with 261 individuals harbouring metacercariae in *A*. *balthica*, *G*. *acronicus* and *P*. *casertanum/Sphaerium* sp. (overall prevalence 10.5%; Table 1). Prevalence was similar in the two gastropods (11.1 %) but lower in sphaeriid clams (5.8%; Supplementary Table S1). No metacercarial infections were detected in *Valvata* sp., and multiple infections were not observed in any of the molluscan hosts (Table 1 and Supplementary Table S1). In total, 11 metacercarial isolates were successfully identified molecularly (4.2 % of all 261 infected molluscs; Table 1).

### 3.1 Diversity and phylogeny of sampled trematodes

Molecular phylogenetic analyses revealed 24 trematode species and species-level lineages (hereinafter referred to as “species”) belonging to seven families in five molluscan hosts recorded in 2024 (see detailed molecular results below; Table 2). These include four schistosomatid lineages previously characterised molecularly in the lake (see details, including photomicrographs of cercariae in Fig. 4A–F, 5J–L, 6G–I in Kibet et al. (in press)). An additional unidentified strigeid (here provisionally named Strigeidae gen. sp.) was detected in *Valvata* sp. (Table 2, Fig. 4E and F), but its identity could not be resolved beyond the family level due to limited morphological identification and the absence of successful molecular data. Both key snail species, *A*. *balthica* and *G*. *acronicus*, served as first intermediate hosts for Notocotylidae, Schistosomatidae and Strigeidae representatives and as second intermediate hosts for strigeids, respectively (Table 2).

**Table 2:** Trematodes recorded in molluscan intermediate hosts in Lake Takvatn: comparison of the 2012–2013 survey (Soldánová et al., 2017) and the 2024 survey (present study). First and second molluscan intermediate hosts are shown based on published and newly obtained data. Presence in each survey is indicated by the symbol ×. Original lineage designations from Soldánová et al. (2017) are retained for direct comparison; updated identifications based on recent molecular data are provided in parentheses.

Trematode family/species	First molluscan intermediate host	Second molluscan intermediate host	Soldánová et al. (2017)	Present study
**Family Allocreadiidae**				
*Allocreadium neotenicum*	–a		×	
*Crepidostomum farionis*	*Pisidium casertanum*; *Sphaerium* sp.		×	
*Crepidostomum metoecus*	*Pisidium casertanum*^b^*/Sphaerium* sp.		×	×
*Crepidostomum* sp. 1^c^ (= *Crepidostomum pseudofarionis*)	*Sphaerium* sp.		×	
*Crepidostomum* sp. 2^c^ (= *Crepidostomum brinkmanni*)	–a		×	
**Family Diplostomidae**				
*Diplostomum phoxini*	*Ampullaceana balthica*		×	
*Diplostomum* sp. Lineage 3^c^	*Ampullaceana balthica*^d^		×	
*Diplostomum* sp. Lineage 4^c^ (= *D*. *petromyzifluviatilis*)	*Ampullaceana balthica*		×	×
*Diplostomum* sp. Lineage 5^c^	*Ampullaceana balthica*^d^		×	
*Diplostomum* sp. Lineage 6^c^	*Ampullaceana balthica*		×	×
*Tylodelphys* sp.^c^	*Ampullaceana balthica*^d^		×	
**Family Echinostomatidae**				
*Echinoparyphium recurvatum*	*Ampullaceana balthica*	*Pisidium casertanum/Sphaerium* sp.	×	×
**Family Notocotylidae**				
*Notocotylus ikutai*^e^	*Ampullaceana balthica*; *Gyraulus acronicus*	–f	×	×
*Notocotylus* sp. 1^g^	*Ampullaceana balthica*	–f		×
*Notocotylus* sp. 2^g^	*Gyraulus acronicus*	–f		×
**Family Plagiorchiidae**				
*Plagiorchis* sp. 1^c^	*Ampullaceana balthica*		×	×
*Plagiorchis* sp. 2^c^	*Ampullaceana balthica*		×	×
*Plagiorchis* sp. 3^c^	*Ampullaceana balthica*		×	×
*Plagiorchis* sp. 4^c^	*Ampullaceana balthica*		×	×
*Plagiorchis* sp. 5^c^	*Ampullaceana balthica*		×	×
*Plagiorchis* sp. 6^c^	*Ampullaceana balthica*		×	×
*Plagiorchis* sp. 7^c^	*Ampullaceana balthica*		×	×
*Plagiorchis* sp. 11	*Ampullaceana balthica*			×
**Family Schistosomatidae**				
*Trichobilharzia franki* haplotype "peregra" (= *Trichobilharzia* sp. VIII)^h^	*Ampullaceana balthica*	–f	×	×
Avian schistosomatid sp. I6 (= Schistosomatidae gen. sp. X)^h^	*Gyraulus acronicus*	–f		×
Schistosomatidae gen. sp. XXII^g^^,^^h^	*Ampullaceana balthica*	–f		×
Schistosomatidae gen. sp. XXIII^g^^,^^h^	*Gyraulus acronicus*	–f		×
**Family Strigeidae**				
*Apatemon gracilis*	*Ampullaceana balthica*		×	×
*Apatemon* sp. 6	*Gyraulus acronicus*			×
*Apatemon* sp. T1^g^	*Gyraulus acronicus*			×
*Apatemon* sp. T2^g^	*Gyraulus acronicus*			×
*Apatemon* sp.^c^	*Ampullaceana balthica*^d^		×	
*Cotylurus cornutus*	*Ampullaceana balthica*	*Ampullaceana balthica*; *Gyraulus acronicus*	×	×
Strigeidae gen. sp. (unidentified)	*Valvata* sp.			×

a No evidence of molluscs involved in the life cycle.

b *Pisidium casertanum* and *Sphaerium* sp. were not distinguished in the present survey; *P*. *casertanum* was specifically identified as a host in Soldánová et al. (2017).

c Putative new species in Soldánová et al. (2017); *Crepidostomum* sp. 1–2 formally described later by Faltýnková et al. (2020); *Diplostomum* lineages 3–6 discovered in Iceland by Blasco-Costa et al. (2014) based on molecular data.

d First intermediate hosts inferred based on life-cycle data and host specificity in Soldánová et al. (2017).

e Identified as *Notocotylus* sp. from *A*. *balthica* in Soldánová et al. (2017).

f No second intermediate host in the life cycle.

g Putative new species in the present study.

h Updated taxonomic assignment of avian schistosomes according to Kibet et al. (in press); *Trichobilharzia franki* haplotype "peregra" discovered in Iceland by Jouet et al. (2010) based on molecular data; Avian schistosomatid sp. I6 discovered in the Czech Republic by Aldhoun et al. (2012) based on molecular data.

The most species-rich families in first molluscan intermediate hosts were Plagiorchiidae (eight species), followed by Strigeidae (five putative species) and Schistosomatidae (four species; Kibet et al., in press) (Table 2). *Ampullaceana balthica* harboured the highest trematode diversity, hosting the majority of detected taxa across six families (Table 2). The most prevalent families in this host were Plagiorchiidae (6.7%), followed by Strigeidae (3.5%) and Diplostomidae (2.6%), with a comparable prevalence in the remaining families Schistosomatidae, Notocotylidae and Echinostomatidae (1.4–1.8%; Supplementary Table S1). In contrast, *G*. *acronicus* supported lower overall diversity spanning three trematode families, comprising two schistosomatid, two strigeid and one notocotylid species (Table 2), the latter two being the most prevalent families in this host (1.0%) (Supplementary Table S1). Sphaeriid clams were infected exclusively by one allocreadiid, while *Valvata* sp. yielded only a single unidentified strigeid infection (Table 2).

Only two trematode families, each represented by a single species, were detected in molluscs serving as second intermediate hosts. Metacercariae of *Cotylurus cornutus* (Strigeidae) were found in both *A. balthica* and *G. acronicus*, with similar prevalence (11.4% and 10.4%, respectively), while sphaeriid clams were infected exclusively by *Echinoparyphium recurvatum* (Echinostomatidae) (5.8%) (Table 1 and Supplementary Table S1). The infection intensity of metacercariae varied between the two host species. In *A. balthica*, intensity ranged from 1 to 50 metacercariae (mean = 4.0 ± 5.1) and nearly half of infected snails (48.6%) harboured only 1–2 metacercariae, while high intensities (15–50 metacercariae per snail) were rare (0.6% of all cases). In *G. acronicus*, infection intensity ranged from 1 to 25 metacercariae (mean = 2.5 ± 3.5), with most infected snails (78.1%) harbouring 1–2 metacercariae and higher intensities (11–25 metacercariae per snail) occurring infrequently (1.4% of all cases). In sphaeriid clams infected with *E. recurvatum*, intensity ranged from 1 to 5 metacercariae (mean = 2.6 ± 1.5).

Phylogenetic reconstructions were used to assess species diversity within the collected genera, or within the family in the case of notocotylids. For most taxa, individual results are presented below, organised by genus (and by the family Notocotylidae). The genus *Crepidostomum* is an exception, as rediae were recovered from only two *P*. *casertanum/Sphaerium* sp. clam specimens and were unequivocally identified as *C. metoecus* (photomicrographs of cercariae not available) based on partial COI gene sequences. Therefore, a phylogenetic reconstruction was not performed for this species. Results for avian schistosome lineages (Table 2) from Lake Takvatn are presented in Kibet et al. (in press), including photomicrographs of cercariae (shown in Fig. –F, 5J–L, 6G–I in Kibet et al., in press).

#### 3.1.1 *Plagiorchis* (Plagiorchiidae)

**Fig. 1:**
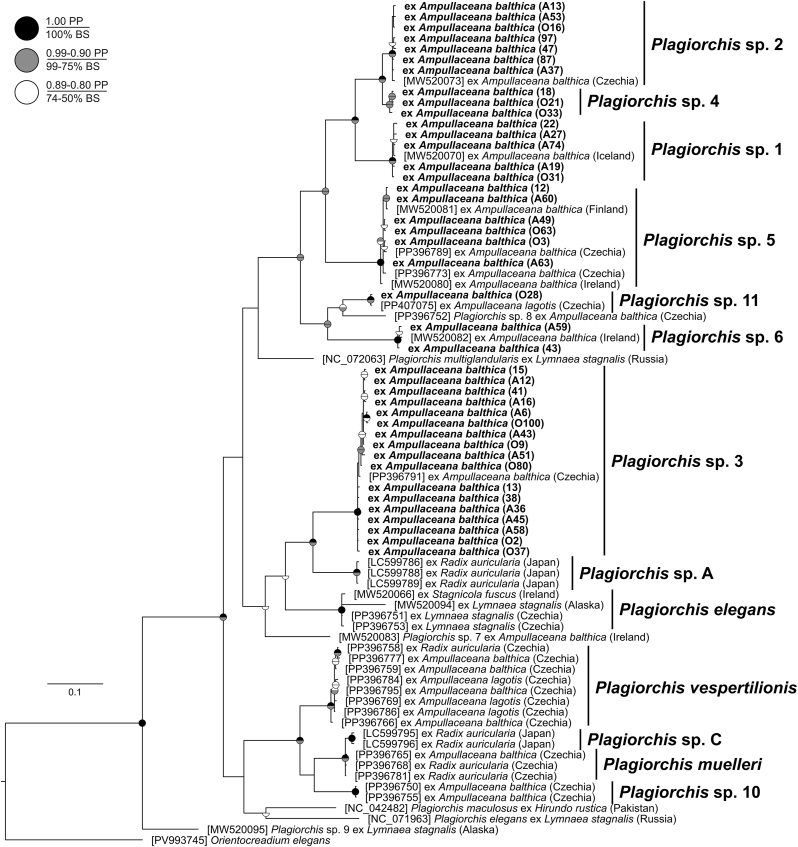
Phylogenetic tree of 78 sequences representing various *Plagiorchis* species reconstructed using Bayesian inference. The tree is based on a 741 bp-long alignment of the partial COI gene region and is rooted with *Orientocreadium elegans* as the outgroup. Circles at the nodes indicate posterior probabilities (upper half) and bootstrap support values (lower half) according to the legend in the upper corner. Branch lengths represent the number of substitutions per site. Newly generated sequences are shown in bold. Parentheses indicate either isolate numbers (for newly generated sequences) or countries of origin (for sequences retrieved from GenBank).

**Fig. 2:**
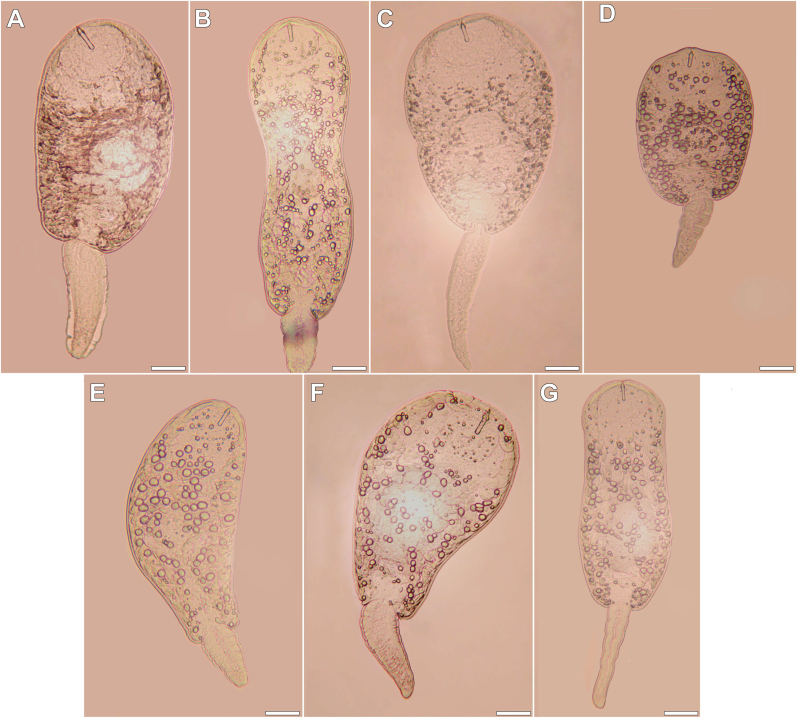
Photomicrographs of live cercariae of *Plagiorchis* spp. (Plagiorchiidae) ex *Ampullaceana balthica*. (A) *Plagiorchis* sp. 1 *sensu*Soldánová et al. (2017); (B) *Plagiorchis* sp. 2 *sensu*Soldánová et al. (2017); (C) *Plagiorchis* sp. 3 *sensu*Soldánová et al. (2017); (D) *Plagiorchis* sp. 4 *sensu*Soldánová et al. (2017); (E) *Plagiorchis* sp. 5 *sensu*Soldánová et al. (2017); (F) *Plagiorchis* sp. 6 *sensu*Soldánová et al. (2017); (G) *Plagiorchis* sp. 11 *sensu*Kundid et al. (2024). Photomicrograph of *Plagiorchis* sp. 7 *sensu* Soldánová et al. (2017) was not available. Scale-bars: 50 μm.

The final alignment for assessing *Plagiorchis* species diversity was constructed from 78 partial COI *Plagiorchis* sequences encompassing 18 species/lineages (with the addition of the *Orientocreadium elegans* ortholog as an outgroup), and spanned 741 unambiguously aligned nucleotide positions. Both phylogenetic analyses (BI and ML) generated trees with congruent topologies, and therefore only the BI tree is presented with posterior probabilities and bootstrap support values (Fig. 1). The phylogenetic tree unequivocally confirmed the presence of seven *Plagiorchis* species, including six species *sensu* Soldánová et al. (2017) (*Plagiorchis* sp. 1–6; Fig. 2A–F) and one *sensu* Kundid et al. (2024) (*Plagiorchis* sp. 11; Fig. 2G) in *A*. *balthica* in Lake Takvatn. However, the sequence of *Plagiorchis* sp. 7 from a single snail specimen was not included in the phylogenetic analyses due to insufficient sequence length (photomicrographs of cercaria not available). Except for *Plagiorchis* sp. 7, only minor intraspecific variability was recorded among conspecific specimens.

#### 3.1.2 *Apatemon* (Strigeidae)

**Fig. 3:**
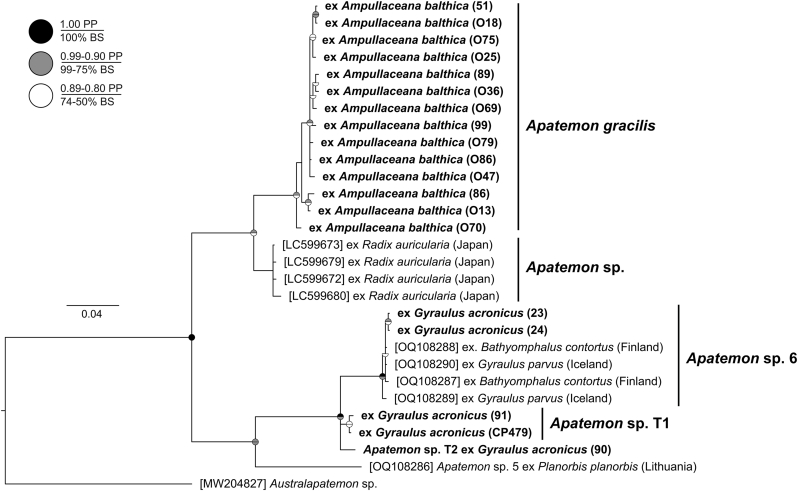
Phylogenetic tree of 28 sequences representing various *Apatemon* species reconstructed using Bayesian inference. The tree is based on a 729 bp-long alignment of the partial COI gene region and is rooted with *Australapatemon* sp. as the outgroup. Circles at the nodes indicate posterior probabilities (upper half) and bootstrap support values (lower half) according to the legend in the upper corner. Branch lengths represent the number of substitutions per site. Newly generated sequences are shown in bold. Parentheses indicate either isolate numbers (for newly generated sequences) or countries of origin (for sequences retrieved from GenBank).

**Fig. 4:**
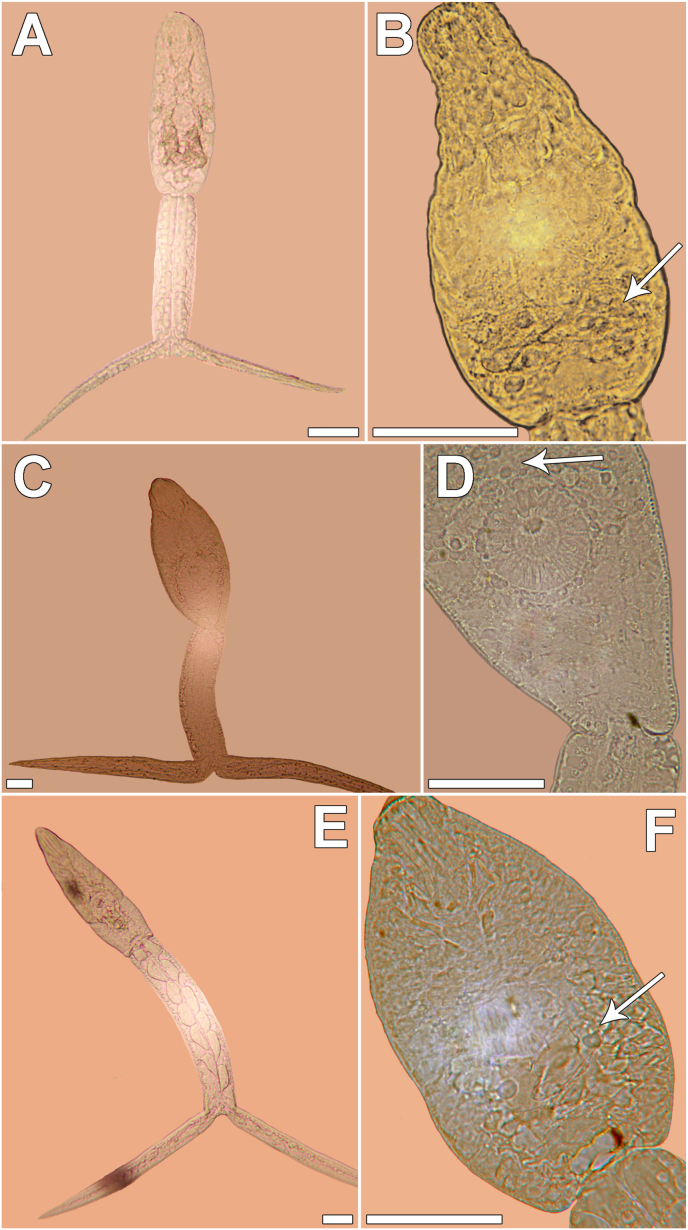
Photomicrographs of live cercariae of the family Strigeidae: (A–B) *Apatemon gracilis* ex *Ampullaceana balthica*; (C–D) *Cotylurus cornutus* ex *A. balthica*; (E–F) Strigeidae gen. sp. ex *Valvata* sp. (not molecularly identified). (A, C, E) General view of cercaria, scale-bar 100 μm; (B, D, F) Detail of body, arrows indicate the position of penetration glands, scale-bar 50 μm.

**Fig. 5:**
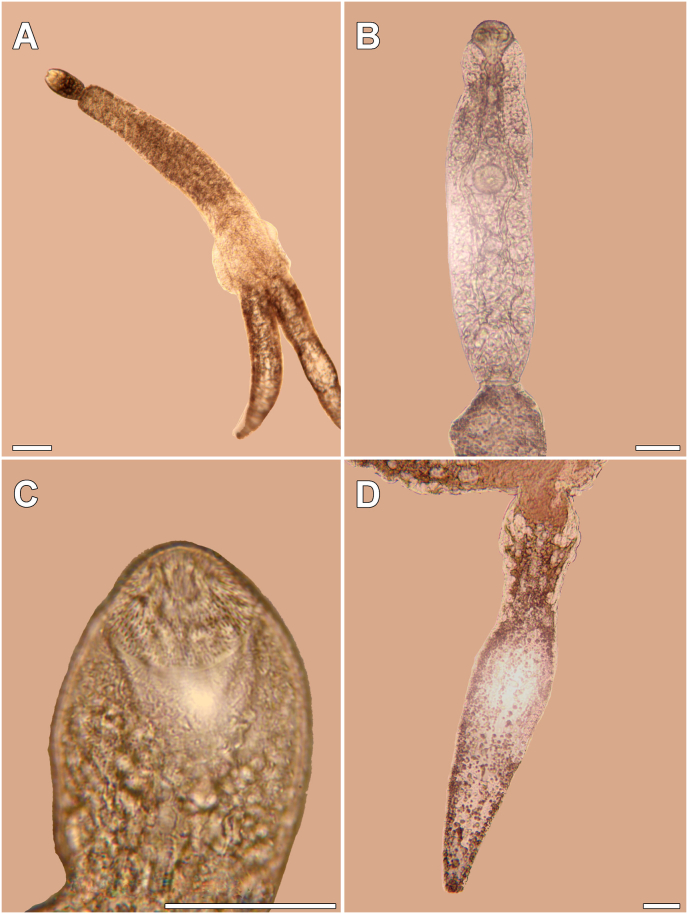
Photomicrographs of live cercariae of *Apatemon* sp. 6 *sensu*Faltýnková et al. (2023) (Strigeidae) ex *Gyraulus acronicus*. (A) General view of cercaria; (B) Detail of body; (C) Anterior body region with oral spines; (D) Detail of furca. Scale-bars: (A) 100 μm; (B–D) 50 μm.

**Fig. 6:**
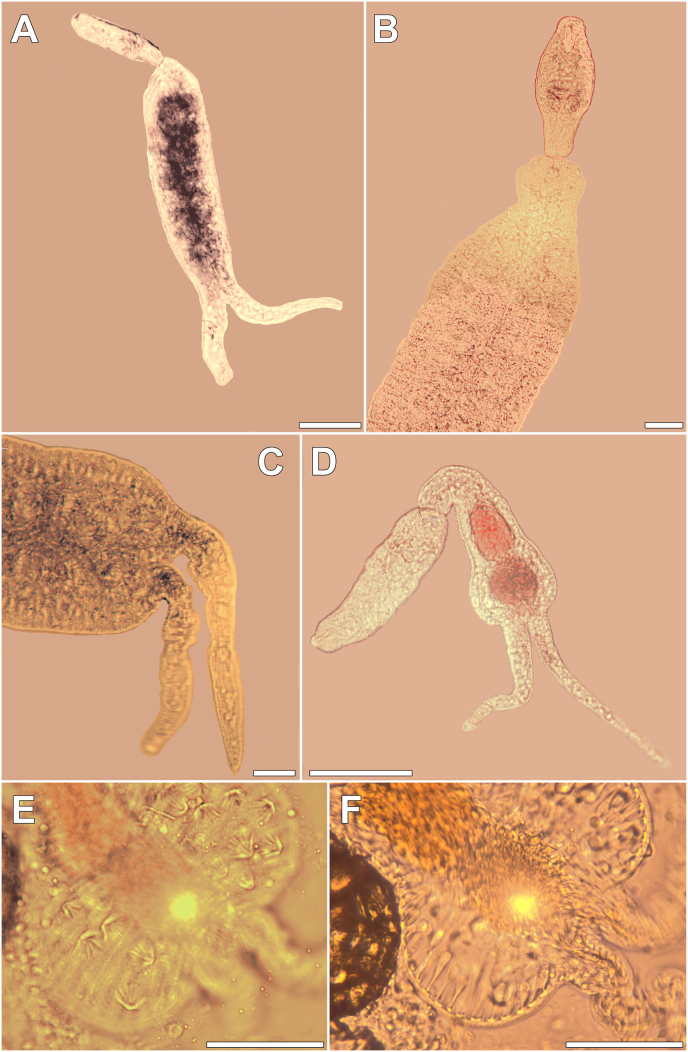
Photomicrographs of live cercariae of *Apatemon* spp. (Strigeidae) ex *Gyraulus acronicus*. (A–C) *Apatemon* sp. T1: (A) General view of cercaria; (B) Detail of body; (C) Detail of furcae. (D–F) *Apatemon* sp. T2: (D) General view of cercaria; (E–F) Detail of bulbus region. Scale-bars: (A, D) 100 μm; (B, C, E, F) 50 μm.

The final alignment for assessing *Apatemon* species diversity was constructed from 28 partial COI *Apatemon* sequences encompassing six species/lineages (with the addition of the *Australapatemon* sp. ortholog as an outgroup), and spanned 729 unambiguously aligned nucleotide positions. Both phylogenetic analyses (BI and ML) generated trees with congruent topologies, and therefore only the BI tree is presented with posterior probabilities and bootstrap support values (Fig. 3). The phylogenetic tree revealed the presence of four *Apatemon* taxa among the collected specimens (Fig. 4, Fig. 5, Fig. 6). The most prevalent species among all detected strigeids (including *Cotylurus cornutus*; see below) was *A. gracilis* (Fig. 4A and B), infecting only *A*. *balthica* in the lake (prevalence not calculated, but the majority of isolates belonged to this species, n = 52 of a total of 56; Supplementary Table S1). This species also exhibited minor intraspecific genetic variability, and although no congeneric sequences were available in GenBank for the COI region used for tree building, the other amplified COI region (using primers from Moszczynska et al., 2009) confirmed the species identity of collected specimens. The second species was *Apatemon* sp. 6 *sensu* Faltýnková et al. (2023) which was detected only in three *G*. *acronicus* snail individuals (Fig. 5). Additionally, two *G. acronicus* were infected with *Apatemon* sp. T1 and one *G. acronicus* with *Apatemon* sp. T2 (Fig. 6A–C and Fig. 6D–F, respectively). These two species exhibited substantial genetic divergence from each other in the analysed partial COI region (1.92% difference across 729 bp) and also differed morphologically (Fig. 6). Phylogenetic analyses suggested their sister position relative to *Apatemon* sp. 6. The ITS1–5.8S–ITS2 sequence obtained from a single specimen of *G. acronicus* (isolate CP479) was identical to the orthologous sequence of *Apatemon* sp. 6 deposited in GenBank (OQ102389). Uncorrected *p*-distance of the two *Apatemon* species (i.e., *Apatemon* sp. T1, isolate 91 and *Apatemon* sp. T2, isolate 90) from collected *Apatemon* sp. 6 (isolates 23 and 24) was in both cases 3.98%.

#### 3.1.3 *Cotylurus* (Strigeidae)

**Fig. 7:**
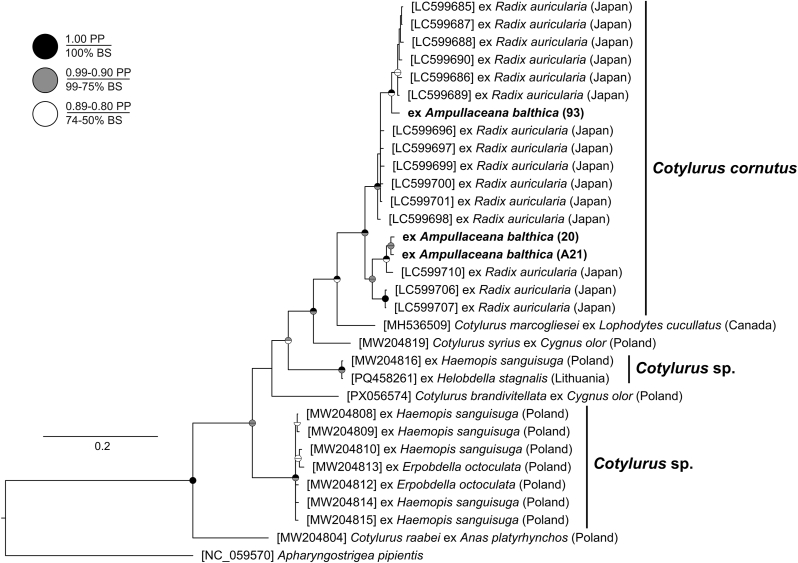
Phylogenetic tree of 31 sequences representing various *Cotylurus* species reconstructed using Bayesian inference. The tree is based on a 793 bp-long alignment of the partial COI gene region and is rooted with *Apharyngostrigea pipientis* as the outgroup. Circles at the nodes indicate posterior probabilities (upper half) and bootstrap support values (lower half) according to the legend in the upper corner. Branch lengths represent the number of substitutions per site. Newly generated sequences are shown in bold. Parentheses indicate either isolate numbers (for newly generated sequences) or countries of origin (for sequences retrieved from GenBank).

The final alignment for assessing *Cotylurus* species diversity was constructed from 31 partial COI *Cotylurus* sequences encompassing seven species *sensu* Stanevičiūtė et al. (2025) (with the addition of the *Apharyngostrigea pipientis* ortholog as an outgroup), and spanned 793 unambiguously aligned nucleotide positions. Both phylogenetic analyses (BI and ML) generated trees with congruent topologies, and therefore only the BI tree is presented with posterior probabilities and bootstrap support values (Fig. 7). The phylogenetic tree revealed the presence of a single species, *C. cornutus*, in *A*. *balthica* (Fig. 4C and D); however, a substantial degree of intraspecific genetic variability was observed among conspecific specimens.

#### 3.1.4 *Diplostomum* (Diplostomidae)

**Fig. 8:**
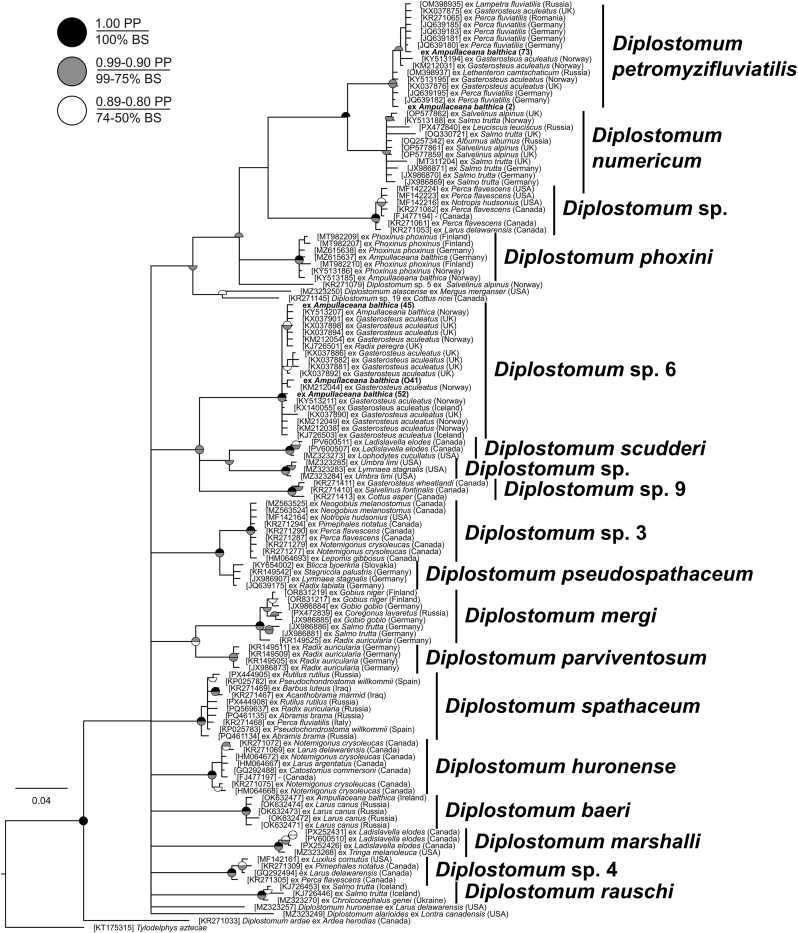
Phylogenetic tree of 135 sequences representing various *Diplostomum* species reconstructed using Bayesian inference. The tree is based on a 356 bp-long alignment of the partial COI gene region and is rooted with *Tylodelphys aztecae* as the outgroup. Circles at the nodes indicate posterior probabilities (upper half) and bootstrap support values (lower half) according to the legend in the upper corner. Branch lengths represent the number of substitutions per site. Newly generated sequences are shown in bold. Parentheses indicate either isolate numbers (for newly generated sequences) or countries of origin (for sequences retrieved from GenBank).

**Fig. 9:**
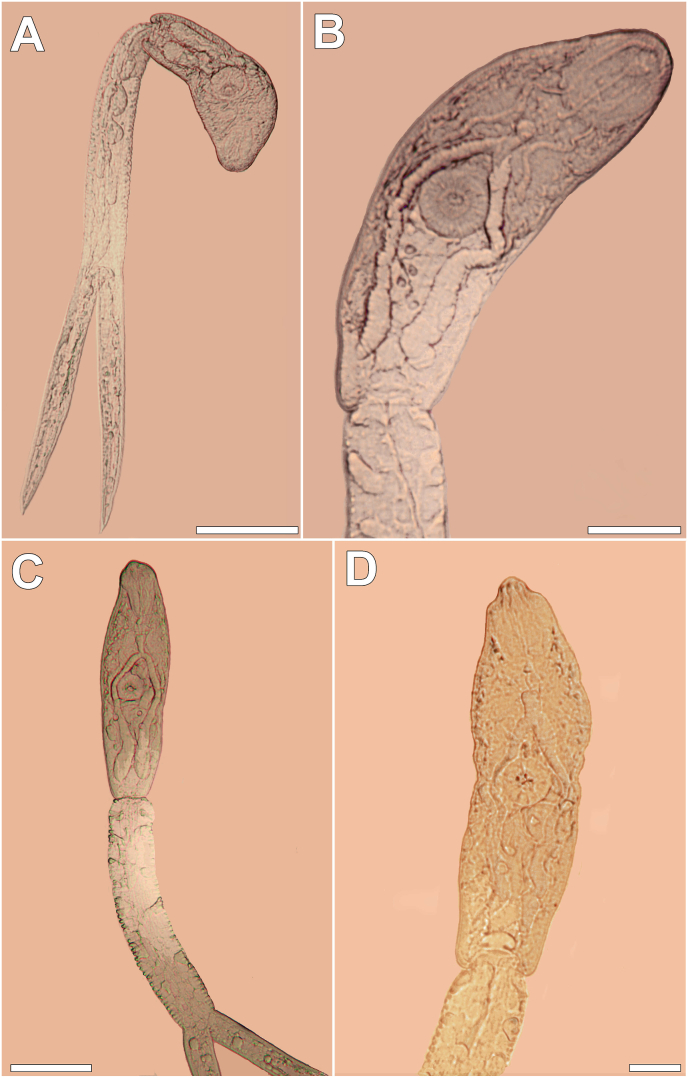
Photomicrographs of live cercariae of *Diplostomum* spp. (Diplostomidae) ex *Ampullaceana balthica*. (A–B) *Diplostomum* sp. Lineage 4 *sensu*Blasco-Costa et al. (2014) (syn. *D. petromyzifluviatilis*); (C–D) *Diplostomum* sp. Lineage 6 *sensu*Blasco-Costa et al. (2014). (A, C) General view of cercaria, scale-bar 100 μm; (B, F) Detail of body, scale-bar 50 μm.

The final alignment for assessing *Diplostomum* species diversity was constructed from 135 partial COI *Diplostomum* sequences encompassing 23 species/lineages *sensu* Blasco-Costa et al. (2014), Faltýnková et al. (2014), Soldánová et al. (2017) and Lebedeva et al. (2022) (with the addition of the *Tylodelphys aztecae* ortholog as an outgroup), and spanned 356 unambiguously aligned nucleotide positions. Both phylogenetic analyses (BI and ML) generated trees with congruent topologies, and therefore only the BI tree is presented with posterior probabilities and bootstrap support values (Fig. 8). The phylogenetic analyses revealed the presence of two *Diplostomum* species in *A*. *balthica* in Lake Takvatn: *Diplostomum petromyzifluviatilis* (following Lebedeva et al., 2022), previously identified as *Diplostomum* Lineage 4 *sensu* Blasco-Costa et al. (2014) (Fig. 9A and B), and *Diplostomum* sp. LIN 6 *sensu* Blasco-Costa et al. (2014) (Fig. 9C and D). Minor intraspecific genetic variability was observed among conspecific specimens.

#### 3.1.5 *Echinoparyphium* (Echinostomatidae)

**Fig. 10:**
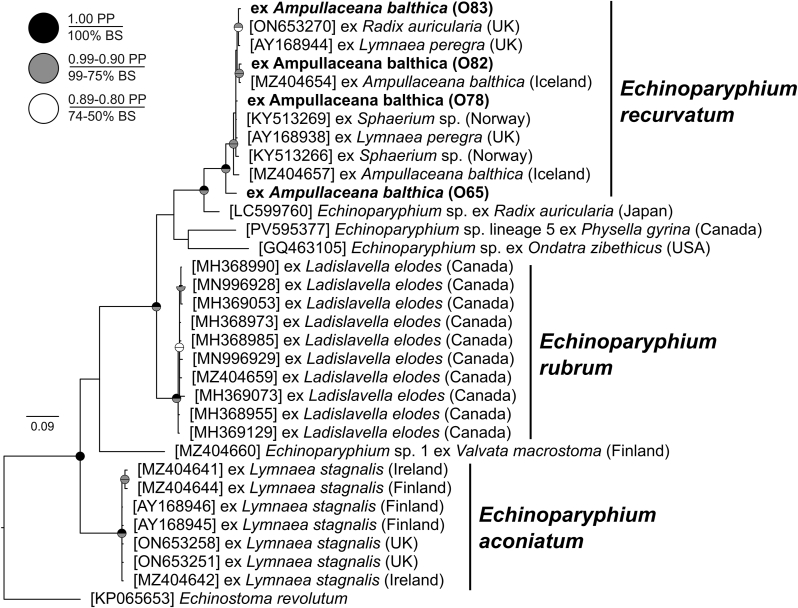
Phylogenetic tree of 32 sequences representing various *Echinoparyphium* species reconstructed using Bayesian inference. The tree is based on a 425 bp-long alignment of the partial ND1 gene region and is rooted with *Echinostoma revolutum* as the outgroup. Circles at the nodes indicate posterior probabilities (upper half) and bootstrap support values (lower half) according to the legend in the upper corner. Branch lengths represent the number of substitutions per site. Newly generated sequences are shown in bold. Parentheses indicate either isolate numbers (for newly generated sequences) or countries of origin (for sequences retrieved from GenBank).

**Fig. 11:**
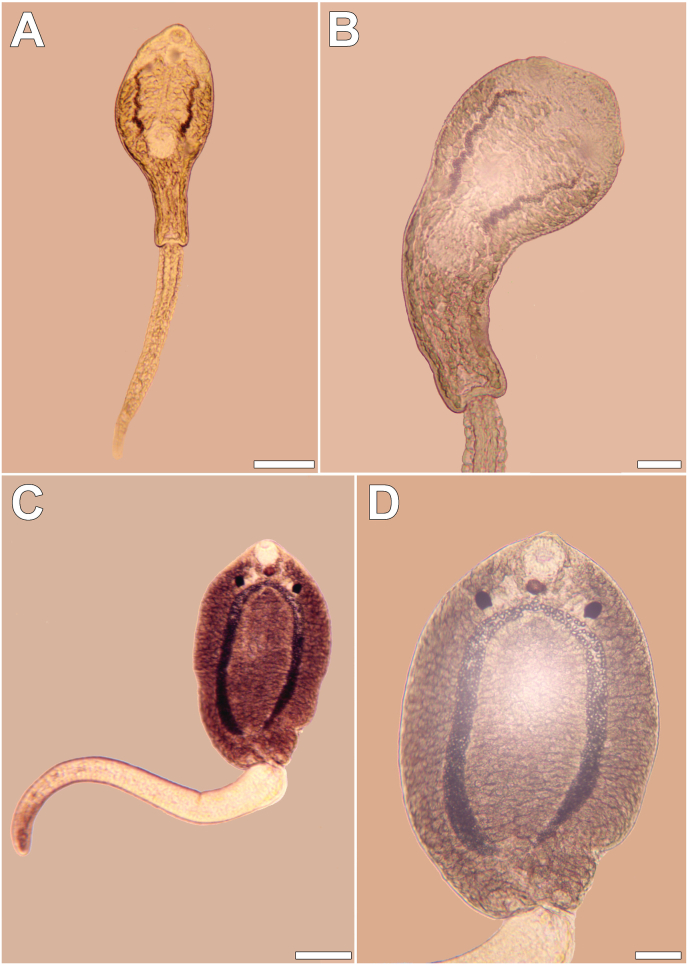
Photomicrographs of live cercariae of families Echinostomatidae and Notocotylidae. (A–B) *Echinoparyphium recurvatum* ex *Ampullaceana balthica*; (C–D) *Notocotylus ikutai* ex *Gyraulus acronicus*. (A, C) General view of cercaria, scale-bar 100 μm; (B, F) Detail of body, scale-bar 50 μm.

The final alignment for assessing *Echinoparyphium* species diversity was constructed from 32 partial ND1 *Echinoparyphium* sequences encompassing seven species (with the addition of the *Echinostoma revolutum* ortholog as an outgroup), and spanned 425 unambiguously aligned nucleotide positions. Both phylogenetic analyses (BI and ML) generated trees with congruent topologies, and therefore only the BI tree is presented with posterior probabilities and bootstrap support values (Fig. 10). All collected *Echinoparyphium* specimens were identified as *E. recurvatum* (Fig. 11A and B) and exhibited minor to substantial intraspecific variability. Two specimens collected from *A. balthica* (isolates O65 and A38 (the latter not shown in the tree)) were genetically divergent from the rest and occupied a basal position to all other *E. recurvatum* conspecifics in phylogenetic analyses.

#### 3.1.6 Notocotylidae

**Fig. 12:**
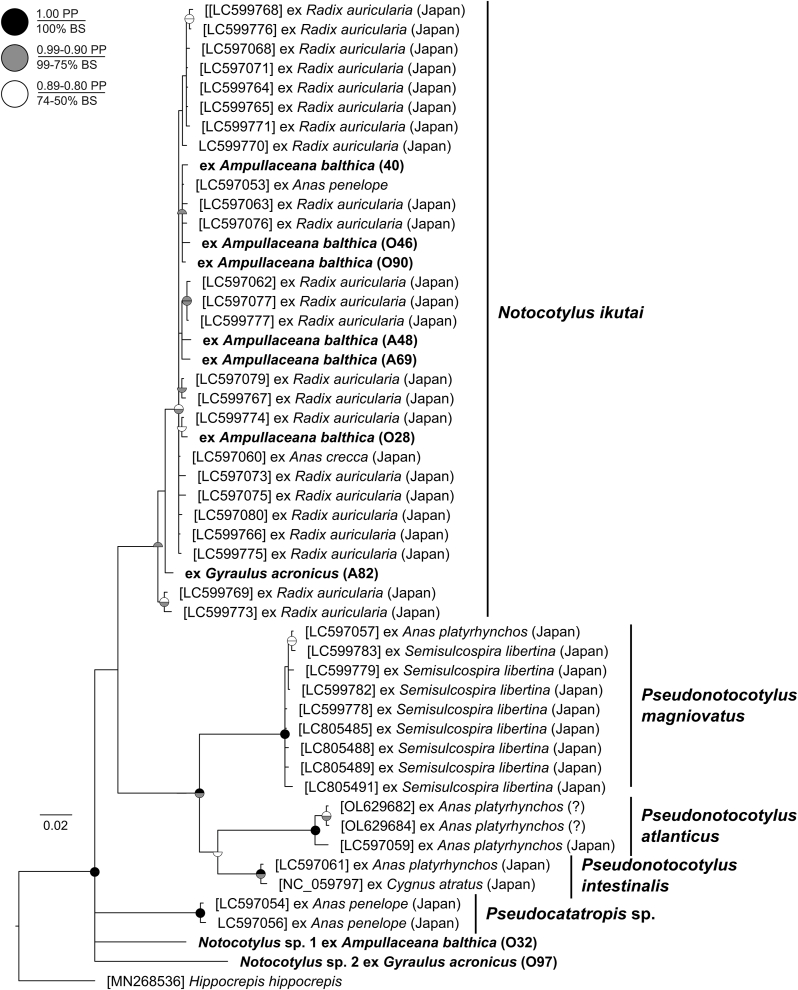
Phylogenetic tree of 51 sequences representing various notocotylid taxa reconstructed using Bayesian inference. The tree is based on a 723 bp-long alignment of the partial COI gene region and is rooted with *Hippocrepis hippocrepis* as the outgroup. Circles at the nodes indicate posterior probabilities (upper half) and bootstrap support values (lower half) according to the legend in the upper corner. Branch lengths represent the number of substitutions per site. Newly generated sequences are shown in bold. Parentheses indicate either isolate numbers (for newly generated sequences) or countries of origin (for sequences retrieved from GenBank).

The final alignment for assessing notocotylid species diversity was constructed from 51 partial COI *Notocotylus*, *Pseudonotocotylus*, and *Pseudocatatropis* sequences (*sensu* Izrailskaia et al., 2024) encompassing six species/lineages (with the addition of the *Hippocreppis hippocreppis* ortholog as an outgroup), and spanned 723 unambiguously aligned nucleotide positions. Both phylogenetic analyses (BI and ML) generated trees with congruent topologies, and therefore only the BI tree is presented with posterior probabilities and bootstrap support values (Fig. 12). The majority of molecularly analysed *Notocotylus* specimens were identified as *N. ikutai* (Fig. 11C and D). Only two specimens, one from *A. balthica* and one from *G. acronicus*, were recognised as two phylogenetically divergent species, labelled as *Notocotylus* sp. 1 and *Notocotylus* sp. 2, respectively (Table 2; photomicrographs of cercariae not available). Further determination of the species was hindered by the lack of available notocotylid ortholog sequences in GenBank with proper species identification.

### 3.2 Comparison of trematode species richness and composition between surveys

**Fig. 13:**
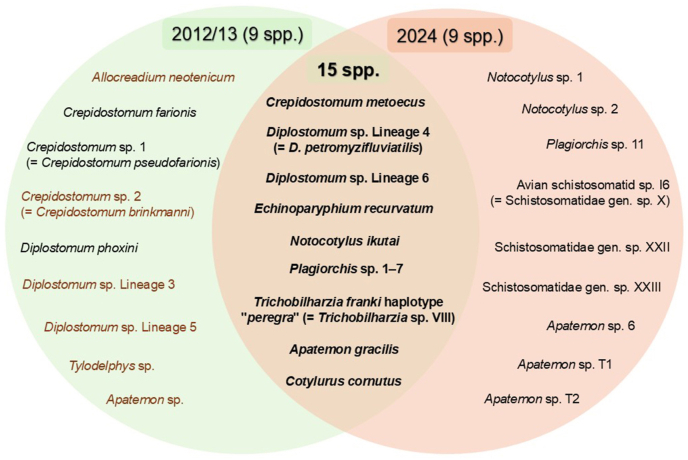
Graphical interpretation of the temporal comparison of molecularly identified trematodes recorded in Lake Takvatn (Norway) between surveys conducted in 2012–2013 (Soldánová et al., 2017) and 2024 (present study), illustrating apparent differences in species composition between surveys based on available records. Taxa shown in brown were inferred to use molluscan first intermediate hosts in the earlier survey based on life cycle evidence, while the remaining taxa were directly observed in molluscs. Strigeidae gen. sp. from the gastropod *Valvata* sp. (present study) is excluded from the diagram (but see Table 2), as its molecular identity and distinctness from other detected taxa could not be confirmed.

Comparison with the molecular survey conducted in 2012–2013 (Soldánová et al., 2017) showed largely consistent trematode diversity at the family level in Lake Takvatn (Fig. 13, Table 2). Soldánová et al. (2017) reported 24 trematode species or species-level genetic lineages from seven families found in multiple arthropod intermediate and fish vertebrate hosts. However, four taxa (*Apatemon* sp., *Diplostomum* sp. Lineage 3, *Diplostomum* sp. Lineage 5 and *Tylodelphys* sp.) were not directly observed in molluscs but were inferred to utilise *A*. *balthica* as the obligatory first intermediate host based on life cycle evidence (Table 2). In addition, two allocreadiid taxa (*Allocreadium neotenicum* and *Crepidostomum* *brinkmanni*) lacked specific molluscan host assignment in the earlier survey (Table 2). However, these species most likely utilise the only two small sphaeriid clams present in the lake as first intermediate hosts, consistent with evidence from other allocreadiid species (Soldánová et al., 2017) and published life cycle data from Europe (Petkevičiūtė et al., 2023). After applying these criteria and excluding the only unresolved strigeid from *Valvata* sp., both surveys comprised 24 mollusc-associated taxa, enabling a temporal comparison of species richness (Fig. 13, Table 2), although this equivalence is partly influenced by differences in how taxa were inferred or excluded between studies.

Despite comparable overall richness, species composition differed between surveys (Fig. 13, Table 2). Fifteen taxa were shared, representing 63% of the trematode fauna previously reported in molluscs by Soldánová et al. (2017). The remaining diversity reflected reciprocal turnover between surveys: nine taxa recorded in 2012–2013 were not detected in 2024, and conversely, nine different taxa detected in the present study were absent from the earlier survey (Fig. 13, Table 2). Six of the newly recorded taxa represent species-level lineages new to science (Kibet et al., in press; present study). However, this apparent equivalence in species richness (24 *vs* 24 taxa) should be interpreted with caution.

In *A. balthica*, five previously reported taxa were not recovered in 2024, including *Diplostomum phoxini*, *Diplostomum* Lineages 3 and 5, *Tylodelphys* sp. and *Apatemon* sp., although these were inferred rather than directly observed in this snail host in the previous study (Soldánová et al., 2017). Conversely, newly recorded taxa included *Notocotylus* sp. 1 (present study) and Schistosomatidae gen. sp. XXII (Kibet et al., in press), while most plagiorchiid lineages persisted across surveys, with only one additional lineage detected (*Plagiorchis* sp. 11) (Fig. 13, Table 2). In *G*. *acronicus*, no infections were recorded in this species acting as first intermediate hosts in the survey by Soldánová et al. (2017), whereas six lineages (two schistosomatids, three strigeids and one notocotylid) were detected in 2024 (Table 2). In addition, *Cotylurus cornutus* metacercariae were recorded at substantially higher prevalence in the present study (73 infected individuals; 10.4% of 701 examined snails; Table 1) compared to the earlier survey, where only two infected individuals were observed (0.6% of 326 individuals examined; Soldánová et al., 2017).

## 4 Discussion

The present study demonstrates high molecular diversity of digenetic trematodes in aquatic snails from Lake Takvatn, with a total of 24 species recorded (including four taxa of avian schistosomes, two of which are putative new genetic lineages, potentially representing new species to science or known species lacking molecular data (Kibet et al., in press) in a subarctic freshwater system. Together with previous findings by Soldánová et al. (2017), this corresponds to a cumulative total of up to 33 trematode taxa recorded in this subarctic system across both surveys, including nine species or species-level lineages recorded only in the earlier survey and not recovered in the present material (Fig. 13). This combined estimate, however, reflects cumulative observations from two independent surveys rather than a single time point. Nevertheless, this relatively high trematode species richness challenges the common assumption that parasite diversity declines toward higher latitudes (Rohde, 1992) and highlights the capacity of subarctic ecosystems to support complex multi-host parasite assemblages. Despite the relatively short ice-free season, low temperatures and simplified host communities characteristic of northern ecosystems, Lake Takvatn harbours a trematode fauna comparable to that reported from more temperate freshwater systems, although such comparisons should be interpreted with caution due to differences in sampling effort, spatial scale, host composition and detection methods among studies. For example, molecular surveys of trematodes across 21 freshwater lakes in Denmark documented 22 trematode species in multiple snail host species (Duan et al., 2021), 36 trematode species were detected in five artificial lakes of the Ruhr River catchment area (Selbach et al., 2020), and 79 trematode species were identified in six lakes in Alberta, demonstrating high parasite diversity also in northern lake ecosystems (Gordy et al., 2020). Similarly diverse trematode assemblages have been found in molluscs, including planorbids, in central European freshwater reserve lakes and temperate reservoir systems (40 species; Schwelm et al., 2021), underscoring that rich trematode communities are not restricted to lower latitudes. In contrast to these multi-lake temperate surveys, the trematode species richness in this study originates from a single lake with a species-poor molluscan community strongly represented by *Ampullaceana balthica*, which alone harboured 71% of the trematode taxa (17 of the 24 species). High parasite diversity therefore does not necessarily require high molluscan diversity or large spatial sampling. Even a single subarctic lake can sustain a rich trematode assemblage when a compatible and abundant intermediate host is present, suggesting that host availability rather than latitude per se may shape trematode diversity patterns in northern and subarctic freshwater ecosystems.

This study is a direct follow-up to the investigation conducted more than a decade after the survey by Soldánová et al. (2017), which reported 24 trematode species or species-level lineages associated with molluscan hosts in Lake Takvatn. Our results also revealed 24 trematode taxa, but although overall species richness remained comparable between surveys, the community composition changed noticeably at the species or lineage level. Approximately one third of previously recorded taxa were not detected (some were reported only from non-molluscan hosts in the earlier survey), while a similar number of new lineages appeared, indicating substantial temporal turnover rather than a net increase or decrease in diversity. These apparent losses and gains should be interpreted with caution, as they may partly reflect incomplete life-cycle sampling as well as differences in detection probability and sampling effort between surveys, rather than definitive absence or presence of taxa. They should also be considered in light of minor differences in inclusion criteria between surveys. This study represents a snapshot resampling approach, providing a rare opportunity to directly compare parasite community composition over a decadal timescale using comparable sampling design and host coverage. Given the general scarcity of temporally comparable datasets on parasite diversity, such replicated studies are essential for distinguishing between community stability and species turnover in natural systems (Hammoud et al., 2026).

In contrast to species/lineage-level comparison, all trematode families persisted across surveys, suggesting long-term stability of higher taxonomic structure despite replacement of individual lineages. The trematode fauna of Takvatn is dominated by bird-transmitted species (only *Crepidostomum metoecus* uses fish as definitive hosts; Soldánová et al., 2017), and variation in the presence, abundance or migratory patterns of definitive bird hosts may strongly influence which lineages are detected in a given period. Surveys in the lake recorded 21 water bird species, including 12 breeding species forming a relatively stable core community, while additional species occur as transient visitors (Klemetsen and Knudsen, 2013). This combination of resident and transient bird species, together with the proximity of Lake Takvatn to major coastal bird areas such as Balsfjord (ca. 20 km), likely increases the diversity and temporal turnover of potential definitive hosts available for parasite transmission. Differences in sampling intensity, large number of sequenced isolates and improved detection of larval stages in our study could also have contributed to the observed turnover. However, these differences were minimised by applying a comparable sampling design, habitat and seasonal coverage and focus on the same most frequently encountered molluscan hosts as in the previous survey by Soldánová et al. (2017), with higher sampling effort in the present study (i.e., 1589 *vs* 667 individuals of *A. balthica* and 701 *vs* 326 of *G. acronicus*). Sampling effort thus reflected the natural composition of the molluscan community, with the most common hosts sampled more intensively. Nevertheless, some variation in sampling effort among less abundant hosts is unavoidable and may influence the detection of rare or low-prevalence trematode taxa, but is unlikely to affect the overall patterns of trematode diversity observed in the key snail host species. Moreover, the amplification and analysis of orthologous genomic markers, as employed in previous studies on the respective taxa, allowed us to assess genetic (and potentially taxonomic) diversity at a comparable level of robustness.

Host-specific patterns further clarify these dynamics. *Ampullaceana balthica* remained the principal transmission hub in the lake system, harbouring the majority of taxa. In contrast, *G. acronicus*, previously of negligible importance as a first intermediate host in Soldánová et al. (2017), harboured taxa belonging to multiple trematode families in the present study and also exhibited a markedly higher occurrence of metacercariae, which may partly reflect the higher number of examined individuals compared to the earlier survey. Rarefaction analyses indicate that trematode diversity associated with *A. balthica* was sampled nearly exhaustively, whereas sampling of *G. acronicus* remained incomplete despite the relatively large number of examined individuals. The diversity of trematodes utilising *G. acronicus* may therefore still be underrepresented, likely reflecting a combination of lower infection prevalence and reduced detection probability in this host. Nevertheless, species richness in Lake Takvatn is unlikely to be an artifact of sampling intensity and may represent a conservative estimate. These results further suggest that the functional role of the two key snail species in transmission networks can change over time, even when overall diversity remains similar. Overall, trematode assemblages in subarctic lakes appear temporally dynamic but structurally persistent, with community patterns governed mainly by species turnover (apparent losses and gains) rather than changes in overall diversity, and strongly influenced by host availability, particularly definitive bird hosts.

This pattern is illustrated by the species of the genus *Plagiorchis*. Previous research revealed seven *Plagiorchis* species in *A*. *balthica* from Lake Takvatn (Soldánová et al., 2017), and all seven were detected again more than 10 years later, with the addition of *Plagiorchis* sp. 11. This lineage was recently characterised by Kundid et al. (2024) from the central European lymnaeid *Ampullaceana lagotis*. Although it represents a newly recognised plagiorchiid lineage, its distribution range and potential dispersal patterns have been associated with highly mobile definitive hosts (Kundid et al., 2024). In the genus *Plagiorchis*, these are typically higher vertebrates (Krasnolobova, 1987; Tkach, 2008). In this case, it is tempting to assume that *Plagiorchis* sp. 11 was introduced to the subarctic lake by migratory birds, such as anatids or scolopacids, which are known to nest and breed at Takvatn (Klemetsen and Knudsen, 2013), or conversely, translocated from higher latitudes to Central Europe by these hosts. This is further supported by the occurrence of some migratory bird species considered potential hosts of *Plagiorchis* spp. in both Central European lakes and Lake Takvatn (e.g., larids; Klemetsen and Knudsen, 2013; Kundid et al., 2024). Nevertheless, *Plagiorchis* sp. 11 was recorded in only three *A. balthica* snails, and since its life cycle has not yet been fully described, it remains speculative whether the trematode was recently introduced to the lake or simply overlooked previously due to its low prevalence. If this species is truly associated only with temporarily present migratory birds, it may be unable to complete its life cycle because of limited definitive host availability (Galaktionov et al., 2019). Most other *Plagiorchis* species (except *Plagiorchis* sp. 4) have also been reported from other northern European countries (Finland, Iceland and Ireland), with their cercariae described morphologically by Kudlai et al. (2021). Our study therefore provides the first evidence of the cercarial morphology of *Plagiorchis* sp. 4.

Our results provide the first evidence that the only planorbid snail present in Lake Takvatn, *G*. *acronicus*, serves as susceptible intermediate hosts for several *Apatemon* taxa. Although planorbids are recognised as common hosts of *Apatemon* in other northern European countries (e.g., Finland, Iceland and Lithuania; Faltýnková et al., 2023), they have not previously been reported as first intermediate hosts of any trematode species in this lake (Soldánová et al., 2017). In comparison with *A. balthica,* only a small proportion of *G*. *acronicus* snails were infected with trematodes (2.3% of 701 examined individuals), which was nevertheless higher than in the earlier survey by Soldánová et al. (2017) and contrasted with the relatively frequent occurrence of metacercariae in this host (10.4%). Similarly to *Plagiorchis*, a higher diversity of *Apatemon* was detected in 2024 than previously (Soldánová et al., 2017), comprising one species (*A*. *gracilis*) congruent with the earlier study and three additional novel species. Two of these newly detected taxa (i.e., *Apatemon* sp. 6 and *Apatemon* sp. T1) exhibited only minor intraspecific genetic variability relative to their conspecifics. *Apatemon* sp. T2 was unfortunately represented by a single specimen, so intraspecific variability could not be assessed. These three species form a well-supported monophyletic group, although their relationship was not fully resolved. Nevertheless, interspecific genetic divergence was apparent (∼2% between *Apatemon* sp. T1 and *Apatemon* sp. T2, and ∼4% between these two species and *Apatemon* sp. 6 (729-bp-long COI sequences). The species distinctiveness is further supported by distinct cercarial morphology. *Apatemon* sp. T1 possesses an elongate, cylindrical body with a conspicuously enlarged tail stem and shorter furcae, whereas *Apatemon* sp. T2 is distinctly pyriform, much shorter, with a bulbous posterior tail stem and longer furcae. The latter species also exhibits a conspicuous orange midbody mass and a dark posterior mass accompanied by star-like tegumental structures on the bulbus. In contrast, *Apatemon* sp. T1 is more translucent, lacks a conspicuous posterior tail structure and has visible spination on the anterior organ (not shown).

The systematics within the genus *Apatemon* remains problematic. Adult stages are morphologically highly uniform and molecular data remain incomplete, particularly the incomplete set of orthologous sequences representing all putative taxa, hindering robust phylogenetic reconstructions (Blasco-Costa et al., 2016; Faltýnková et al., 2023). In addition, some species (i.e., *Apatemon* sp. 1, sp. 2., sp. 3. and sp. 4) are characterised solely by sequence data (Locke et al., 2010), whereas others include morphological information restricted to cercarial stages (i.e., *Apatemon* sp. 5 and sp. 6; Faltýnková et al., 2023). Although COI is a highly variable protein-coding gene widely used in molecular systematics of animals (Avise, 2000), reliance on a single marker may inflate species delimitation, and phylogenetic relationships may vary depending on the gene analysed (Blasco-Costa et al., 2016; Soldánová et al., 2017; Faltýnková et al., 2023). As emphasized by Bray et al. (2022), although molecular data are objective, their interpretation regarding species boundaries is inherently subjective, particularly when molecular-based phylogenetic analyses can yield different topologies depending on the genetic markers used (examples in *Apatemon* by Blasco-Costa et al., 2016; Faltýnková et al., 2023). Consequently, species delimitation thresholds remain unsettled, and mitochondrial COI regions are typically used for species delineation (Blasco-Costa et al., 2016; Soldánová et al., 2017; Faltýnková et al., 2023). This taxonomic issue is evident in our data: *Apatemon* sp. T1 and *Apatemon* sp. T2 are phylogenetically closely related to *Apatemon* sp. 6 (100% similarity in ITS regions and >96% similarity in COI), yet their morphology indicates separate species. Confirmation would require additional molecular markers and examination of adult morphology, which provides key diagnostic characters in trematode taxonomy (Gibson et al., 2002; Cribb et al., 2025). Repeated failure to amplify the orthologous genomic region used in Soldánová et al. (2017) prevented testing whether these two taxa, *Apatemon* sp. T1 and *Apatemon* sp. T2, were previously reported from Lake Takvatn. Nevertheless, at least two of the four detected species are reported from Takvatn for the first time. Given their low prevalence, they may have been overlooked in the previous survey (despite approximately twice as many *G. acronicus* specimens being examined in this study compared to Soldánová et al., 2017) or were introduced by migratory birds, which serve as definitive hosts for *Apatemon* species (e.g., Dubois, 1968; Blasco-Costa et al., 2016; Presswell, 2022), similar to the pattern suggested above for *Plagiorchis*. In the case of recent introductions, these novel trematode findings in Takvatn might indicate higher frequencies or new migration patterns of definitive bird hosts.

In the present study, a single *Cotylurus* species, *C. cornutus*, was identified, exhibiting apparent intraspecific genetic variability. A similar pattern was observed in *Echinoparyphium recurvatum*, both being taxa that utilise molluscs as first and second intermediate hosts. Pronounced mitochondrial structuring within morphologically well-defined *Cotylurus* species has been reported in previous studies, with various monophyletic genetic variants later interpreted as conspecific, i.e., in our study, all collected specimens belong to *C. cornutus* (Pyrka et al., 2022; Stanevičiūtė et al., 2025). Similar substantial intraspecific mitochondrial variability has also been documented in echinostomatids, including *E. recurvatum*, where mitochondrial gene sequence divergence reflects population-level structuring rather than species-level differentiation (Saijuntha et al., 2011a,b; Georgieva et al., 2014; Pantoja et al., 2021). Accordingly, in the absence of consistent morphological or host-associated differences, the genetic diversification observed in our material of *C. cornutus* and *E. recurvatum* most likely represents intraspecific population structure rather than cryptic speciation.

The genus *Diplostomum* was represented by fewer taxa than in the previous survey. Soldánová et al. (2017) reported five taxa associated with *A*. *balthica*, consisting of one described species and four genetic lineages, the latter originally characterised from Iceland by Blasco-Costa et al. (2014), with morphological description of cercariae provided by Faltýnková et al. (2014). In this study, only two were confirmed, namely *Diplostomum* lineage 6 and lineage 4, the latter currently recognised as *D. petromyzifluviatilis* following recent molecular revision (Lebedeva et al., 2022). Lineages 3 and 5 were previously detected only as metacercariae in fish, with *A. balthica* inferred as the first intermediate host, and were not recovered in our material. Since these taxa were never directly observed in snails and likely occurred at low prevalence in the earlier study, their current presence cannot be verified without examination of fish hosts. The species *D. phoxini* was also not detected and was probably incidental in the earlier survey, as its life cycle typically involves cyprinid second intermediate hosts (particularly the specific host *Phoxinus phoxinus*) (Dönges, 1969), which are absent from Lake Takvatn (Amundsen et al., 2009). These findings suggest that the occurrence of *Diplostomum* species in the lake is likely limited by the availability of suitable fish hosts rather than by the absence of competent snail hosts.

A total of three notocotylid taxa were recorded in this study, with *N. ikutai* being the most prevalent and exhibiting substantial genetic variability (seven distinct COI haplotypes were recorded from 11 sequenced specimens). This species was recorded from *A. balthica* in a previous survey by Soldánová et al. (2017), but was recognised only as *Notocotylus* sp. Subsequently, novel sequences for *Notocotylus* obtained from adult specimens parasitising *Anas penelope* in Japan allowed its formal description as *N. ikutai* (Sasaki et al., 2021). The other two species were each recorded in a single snail individual: *Notocotylus* sp. 1 in *A. balthica* and *Notocotylus* sp. 2 in *G. acronicus*. Neither had been previously recorded from Lake Takvatn and, given their very low prevalence, they may have been overlooked or only introduced recently to the lake. Following the revision of the genus *Notocotylus* by Izrailskaia et al. (2024), the family Notocotylidae comprises 15 genera. Although COI sequences are reliable for resolving phylogenetic relationships and species delimitation, the absence of orthologous sequences from congeners prevented precise taxonomic identification of *Notocotylus* sp. 1 and *Notocotylus* sp. 2. Therefore, given their phylogenetic divergence, these two taxa may represent distinct representatives of different notocotylid genera.

Both lymnaeid and planorbid snails served as first intermediate hosts for four species of avian schistosomes in Takvatn, two found in *A. balthica* and two in *G*. *acronicus*. The most prevalent was *Trichobilharzia* sp. VIII *sensu* Kibet et al., in press (syn. *Trichobilharzia franki* haplotype “peregra”), recorded in 28 *A. balthica* snails (1.8% of 1589 individuals of this host). Although this prevalence is slightly higher than typically reported for avian schistosomes in Europe (usually <1%; Soldánová et al., 2013; Horák et al., 2015), it still falls within the expected range and indicates that the taxon is well established in Lake Takvatn. This species was already detected in *A. balthica* by Soldánová et al. (2017) and, although it is widespread across Europe, occurs mainly in northern Europe, including Iceland and Norway (Jouet et al., 2010; Kibet et al., in press). In addition, another genetic lineage unassigned to the nominal species, Schistomatidae gen. sp. X *sensu* Kibet et al., in press, previously reported as Avian schistomatid sp. I6 in the Czech Republic by Aldhoun et al. (2012), was newly detected in *A. balthica*. Only two *G*. *acronicus* snails were infected, each harbouring a different schistosomatid species, Schistostomatidae gen. sp. XXII and Schistosomatidae gen. sp. XXIII, representing new faunistic records from Lake Takvatn (Kibet et al., in press). Avian schistosomes rely on waterfowl as definitive hosts, which are abundant and regularly breed at the lake (Klemetsen and Knudsen, 2013), likely facilitating their persistence and dispersal. The newly detected lineages may therefore represent subarctic-associated species, although their occurrence elsewhere cannot be excluded due to bird-mediated dispersal. Their apparent restriction to northern regions may instead reflect low detection probability, as avian schistosomes typically occur at low prevalence and surveys rarely target small planorbid snails.

## 5 Conclusion

This study provides a decadal snapshot resampling of trematode diversity in Lake Takvatn and represents one of the few assessments of long-term trematode community dynamics in a unique subarctic freshwater system. Unlike the previous survey by Soldánová et al. (2017), our aim was not to reconstruct trematode life cycles but to evaluate changes in species diversity, with emphasis on first intermediate hosts. Our results indicate that a substantial proportion of the *A. balthica* trematode community was consistently detected across surveys, while approximately one third of the trematode community differed compared to the previous assessment more than ten years ago. It remains to be tested whether these changes are due to yearly fluctuations that our snapshot resampling did not capture, or if these dynamics represent longer-term ecological processes, such as changes in the dynamics of migratory bird hosts. Although the presence of larval stages in snails alone does not prove life cycle completion, the repeated detection of most taxa suggests continuous circulation within the system. This relative stability contrasts with the more dynamic parasite assemblages reported from climatically variable temperate regions and likely reflects adaptive responses to the constrained but predictable transmission window typical of subarctic environments (e.g., Nikolaev et al., 2020, 2021, 2023). Importantly, our results also reveal that the planorbid snail *G. acronicus*, previously considered of minor relevance in Soldánová et al. (2017), acts as a competent first intermediate host for multiple trematode species and lineages and participates in transmission pathways as both first and second intermediate host. Its contribution to overall parasite diversity has therefore been underrepresented in earlier study and should be recognised as an integral component of the lake's transmission network. Despite the limited host community, Lake Takvatn supports relatively high trematode diversity comparable to other northern freshwater systems (such as Iceland; Faltýnková et al., 2025), highlighting the ecological significance of the lake as a reservoir of parasite biodiversity. Future monitoring, particularly of less abundant molluscan hosts such as *G. acronicus* and *Valvata* sp., will be essential to better understand host-specific roles and longer-term patterns and stability of trematode community dynamics and transmission processes in subarctic lakes.

## Data availability statement

The sequences generated in this study were deposited in GenBank under the accession numbers shown in the Supplementary data (Supplementary Tables S1–S3). The alignments used for the phylogenetic analysis are available from the corresponding author upon request.

## Financial support

This study was supported by the 10.13039/501100001824Czech Science Foundation (project no. 24-11738S) and the 10.13039/501100010963Institute of Parasitology, Biology Centre of the Czech Academy of Sciences, České Budějovice (10.13039/100013405RVO 60077344).

## CRediT authorship contribution statement

**Michal Benovics:** Conceptualization, Data curation, Formal analysis, Investigation, Methodology, Visualization, Writing – original draft. **Camila Pantoja:** Investigation, Writing – review & editing. **Petra Kundid:** Investigation, Visualization, Writing – review & editing. **Christian Selbach:** Resources, Writing – review & editing. **Miroslava Soldánová:** Conceptualization, Funding acquisition, Investigation, Methodology, Project administration, Resources, Supervision, Validation, Writing – original draft.

## Declaration of competing interest

The authors declare no conflict of interest.
