# Supplementary material for: Trematodes in snail intermediate hosts of a subarctic lake: updated perspectives on molecular diversity
Source: Int J Parasitol Parasites Wildl. 2026 May 14;30:101236. doi: 10.1016/j.ijppaw.2026.101236 (PMC13263629; doi:10.1016/j.ijppaw.2026.101236)
Supplement: Multimedia component 2 [file mmc2.pdf]

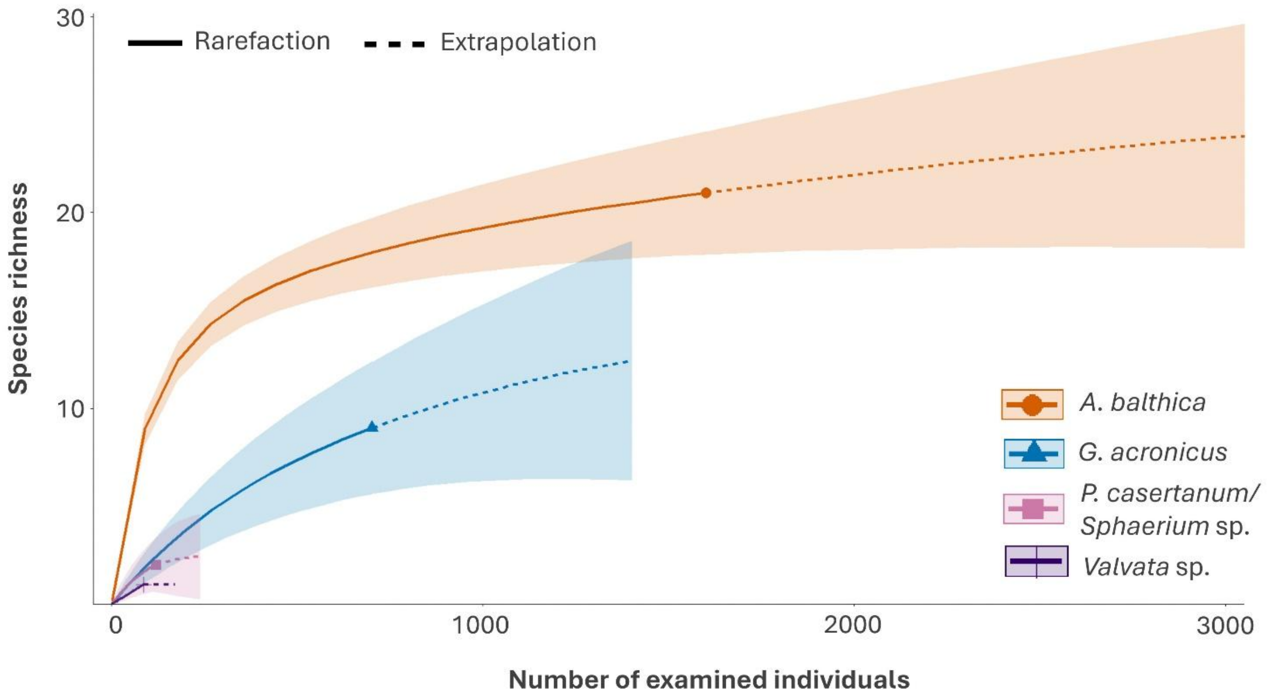

Supplementary Fig. S1. Rarefaction curves of trematode species richness in four snail hosts (*Ampullaceana balthica*, *Gyraulus acronicus*, *Pisidium casertanum*/*Sphaerium* sp. and *Valvata* sp.) based on incidence data. Solid lines represent rarefaction, dashed lines extrapolation and shaded areas 95% confidence intervals.
